# Ethno-racial variations in mental health symptoms among sexually-active gay, bisexual, and other men who have sex with men in Vancouver, Canada: a longitudinal analysis

**DOI:** 10.1186/s12889-024-17743-3

**Published:** 2024-01-24

**Authors:** Seraph L. Bao, Gbolahan Olarewaju, Lu Wang, Jordan Sang, Julia Zhu, Nathan J. Lachowsky, Allan Lal, Aidan Ablona, Darren Ho, Fahmy Baharuddin, Lorenz Villa, Sandy Lambert, Joshun Dulai, David M. Moore

**Affiliations:** 1 Faculty of Medicine, University of British Columbia, Vancouver, BC Canada; 2 BC Centre for Excellence in HIV/AIDS, 608-1081 Burrard Street, Vancouver, BC V6T 1Y6 Canada; 3 University of Victoria, Victoria, BC Canada; 4 Momentum Health Study People of Colour Advisory Board, Vancouver, BC Canada; 5 British Columbia Centre for Disease Control, Vancouver, BC Canada; 6 Community Based Research Centre, Vancouver, BC Canada; 7 Living Positive Resource Centre, Kelowna, BC Canada

**Keywords:** Men who have sex with men, Race, Ethnicity, Mental health, Depression, Anxiety, Minorities

## Abstract

**Background:**

Minority stress from racism and heterosexism may uniquely interact to impact the mental health of racialized sexual minorities. We examined variations in anxiety and depressive symptoms by reported by ethno-racial identity among gay, bisexual, and other men who have sex with men (gbMSM) in Vancouver, Canada.

**Methods:**

We recruited gbMSM aged ≥ 16 years from February 2012 to February 2015 using respondent-driven sampling (RDS). Participants completed computer assisted self-interviews (CASI) at enrollment and every 6 months until February 2017. We examined factors associated with moderate/severe anxiety and depression scores (> 10) on the Hospital Anxiety and Depression Scale (HADS) and differences in key explanatory variables including sociodemographic, psychosocial, and substance use factors. We used multivariable mixed effects models to assess whether moderate/severe scores were associated with ethno-racial identity across all visits.

**Results:**

After RDS-adjustment, of 774 participants, 79.9% of participants identified as gay. 68.6% identified as white, 9.2% as Asian, 9.8% as Indigenous, 7.3% as Latin American, and 5.1% as other ethno-racial identities. Participants contributed a median of 6 follow-up visits (Q1-Q3: 4–7). In the multivariable analysis, Asian participants had decreased odds of moderate/severe anxiety scores compared to white participants (aOR = 0.39; 95% CI: 0.18–0.86), and Latin American participants had decreased odds of moderate/severe depression scores compared to both white (aOR = 0.17; 95% CI: 0.08–0.36) and Asian (aOR = 0.07; 95% CI: 0.02–0.20) participants.

**Conclusion:**

Asian and Latino gbMSM reported decreased mental health symptoms compared to white participants. Asian and Latino gbMSM in Vancouver appear to manage multiple minority stressors without adversely affecting their mental health.

## Key messages

- What is already known on this topic:

◦ Sexual minority groups, including gay, bisexual, and other men who have sex with men (gbMSM), experience higher rates of mental health disorders than their heterosexual counterparts.

◦ Evidence is mixed on how stigma from intersecting minority identities among gbMSM, such as racism experienced by non-white ethno-racial identities, further affects mental health outcomes.

- What this study adds:

◦ Among a sample of urban-residing gbMSM in Vancouver, Canada, ethno-racial minority status was not associated with increased odds of reporting moderate/severe depression or anxiety symptoms.

◦ Asian gbMSM had reduced odds of reported anxiety symptoms and Latino men had reduced odds of depression symptoms compared with white men.

- How this study might affect research, practice, or policy:

◦ More research is needed with ethno-racialized gbMSM to understand mental health risks and vulnerabilities and to qualify survival and resilience narratives surrounding mental health.

◦ Healthcare providers should recognize nuance in how racism and homophobia may uniquely interact to affect the mental health of individual gbMSM.

## Background

Sexual minority groups, including gay, bisexual, and other men who have sex with men (gbMSM), are at greater risk of mental health disorders compared with heterosexual individuals [1–3]. Minority stress theory suggests that these disparities may be explained by the excess stress that individuals from stigmatized groups experience because of their social position [4]. Minority stress may manifest distally (e.g., externalized events such as victimization) or proximally (e.g., internalized social attitudes) [4]. Indeed, research has shown that discrimination, stigma, and internalized homophobia play a role in poor mental health outcomes for gbMSM [5, 6].

However, gbMSM may have multiple marginalized social identities beyond their sexual orientation, including racial or ethnic identity, gender identity, immigration status, socioeconomic status, or HIV serostatus. Indeed, there is extensive literature surrounding the negative impact of racial discrimination on mental health outcomes [7]. Minority identities may be examined through the lens of intersectionality theory, which describes how experiences of oppression from multiple marginalized identities (e.g. racism, heterosexism, classism) interact to produce unique downstream effects [8]. Minority stress theory suggests that sexual minority people of colour experience dual stressors through experiences with both homophobia and racism, also termed the “double jeopardy” hypothesis [9]. However, this diversity of identities can also interact in unique ways, inviting conversations about the contribution of both risk and resilience to mental health [9, 10]. Indeed, many modulating factors, including substance use [11] immigration status [12], may influence mental health outcomes in gbMSM with multiple marginalized identities.

Previous research does not consistently show that people of colour experience more mental health problems compared to their white counterparts, including in sexual minority populations. In the United States (U.S.), the prevalence of mental health disorders is lower in some racial or ethnic minority groups compared with white individuals [13, 14]. Research at the intersection of sexual and ethno-racial minority status is limited, but some U.S. data show that the prevalence of mental health disorders or symptoms is similar or reduced for Black, Latino, and Asian sexual minority individuals compared with white individuals [3, 15–18]. Conversely, some research has shown an increase in suicide attempts for Black [19], Latino [16, 20], and Alaska Native/Pacific Islander [20] sexual minority individuals compared with white individuals. Outside of the U.S., research in the United Kingdom (U.K.) has shown that the impact of minority sexual identity on mental health, as measured by the GHQ-12, is similar along white individuals and people of colour [17]. Conversely, in Australia, non-Anglo-Celtic gay and bisexual men, particularly Indigenous men, were more likely to show symptoms of depression [21].

In Canada, sexual minority individuals experience a higher prevalence of mental health disorders and lower rates of positive mental health than heterosexual individuals [1, 22]. Among all Canadians, Black, South Asian, and Chinese individuals are less likely to have a diagnosed mental health disorder or have suicidal thoughts than their white counterparts [23]. However, Indigenous individuals experience lower rates of complete mental health (emotional well-being; psychological and social functioning) than non-Indigenous individuals [24]. There is comparatively limited recent research outside the U.S. that has centered ethno-racial identity in examining the mental health outcomes of sexual minority groups, and no studies that have investigated outcomes longitudinally. We developed a study to examine the effect of sexual and ethno-racial minority status on symptoms of anxiety and depression in a sample of sexually-active gbMSM in Metro Vancouver, Canada. In doing so, we compare how multiple marginalized identities may uniquely affect mental health outcomes for gbMSM.

## Methods

### Study design

Data for this study were drawn from the Momentum Health Study, a bio-behavioural prospective cohort study investigating sexual, psychosocial, and substance use patterns among sexually-active gbMSM living in Metropolitan Vancouver, Canada. Participants were recruited from February 2012 to February 2015 using respondent-driven sampling (RDS) [25], a formalized chain-referral sampling methodology that leverages the social networks of participants to recruit from minority or marginalized populations [26]. RDS offers some advantages over more traditional sampling strategies such as facility-based sampling, time-location sampling, snowball sampling or targeted sampling for these populations, but comes with its own limitations [27].

Participant eligibility included: gender identity as a man, regardless of sex assigned at birth; age 16 years or older; reported sex with another man in the past 6-months (P6M); current residence in Metro Vancouver; and ability to complete the questionnaire in English. Initial seeds were recruited via word of mouth through community partners and later through advertisements on gbMSM-targeted websites and mobile phone applications. After enrollment, all participants were provided with up to six vouchers and asked to recruit other eligible gbMSM from their social networks. We obtained written informed consent and participants completed 90-min in-person study visit at enrollment. Participants were also given the option of participating in follow-up visits every 6 months for a maximum of four years. At all study visits, participants completed a computer-assisted, self-administered interview (CASI) which asked about socio-demographic, psychosocial, and behavioural factors, including sexual health and substance use. Participants also met with a study nurse for serologic testing for HIV, syphilis, and hepatitis C. The nurse also asked about history of diagnosed mental health and substance use disorders. Study participants received $50 CAD for each study visit and an additional $10 CAD for each eligible recruit. Data for this analysis were collected until February 2017. More detailed study procedures are published elsewhere [28].

### Patient and public involvement

All study participants were directly recruited into the study as seed participants or were recruited by participants already in the study. The Momentum Study is guided by a Community Advisory Board, made of 4 – 8 members of the GBM community and community-based organizations in Vancouver which met on a quarterly basis. The research questions and analysis plan for this paper were guided by a specific advisory committee, the Momentum Health Study People of Colour Advisory Board, composed of members of the community and the research team who identify as people of colour. All members were offered authorship on the paper so as to understand the results and assist in interpretation. All conference presentations and published manuscripts from the Momentum Study are made available on the study’s website (https://momentumstudy.ca/journal-articles-and-summaries) for study participants to access. We also provide plain language summaries of all published manuscripts.

### Variables of interest

Participants were asked to identify the ethnic or racial group they most identified with from a list of 14 possibilities: Indigenous, White, Chinese or Taiwanese, Japanese, Korean, Filipino, South Asian, Southeast Asian, West Asian, Arab, Latino, Black, Pacific Islander, or Other. If “Other” was selected, participants could enter their preferred identity in free text. Participants could also select other ethno-racial groups they identified with in addition to the group they most identified with. These categories were then grouped into five larger ethno-racial groups: white, Indigenous, Latino, Asian and Other for analysis. The Hospital Anxiety and Depression Scale (HADS) [29] was used to ascertain current symptoms of anxiety and depression. Participants provided responses using 4-point scales to statements designed to assess anxiety and depression (e.g. “I get a sort of frightened feeling as if something awful is about to happen”) [29]. Scores for each subscale (anxiety and depression independently) range from 0 to 21 and are categorized as: normal (0–7), mild [8–10], and moderate/severe (> 10) [29]. The HADS scale has been widely used in both research and clinical settings to measure clinically significant symptoms of anxiety and depression and appears to perform as well as other measures such as the Beck’s Depression Index and the Personal Health questionnaire [30]. The Cronbach’s α for anxiety (0.84) and depression (0.79) scores measured at enrollment indicating good internal consistency.

Sociodemographic variables included age, income, and education, sexual identity, immigration status, self-reported HIV serostatus, and being “out’ with their sexual orientation. In addition to the HADS, other psychological factors were measured with the Gay/Bisexual Self-Esteem Scale [31], Lubben Social Network Scale [32], and Loneliness Scale for Emotional and Social Loneliness [33]. As substance use has been shown to be associated with mood and anxiety symptoms [34, 35], participants also completed the Alcohol Use Disorder Identification Test (AUDIT) [36] and were asked about their substance use patterns over the P6M including their use of tobacco, cannabis, crystal methamphetamine, stimulants, depressants, opiates, and hallucinogens.

### Statistical analysis

We calculated RDS-I adjusted descriptive statistics for data collected at enrollment and bivariate analyses comparing variables across the five ethno-racial identities. We used Chi square or Fisher’s exact tests to compare categorical variables and Kruskal–Wallis tests for continuous variables.

We used three-level generalized linear mixed models (GLMM) to examine associations with moderate/severe scores (defined as scores > 10) on the HADS anxiety and depression subscales, at each study visit including enrollment and all available follow-up visits. These models used the RDS chain as the first level cluster to account for biases associated with recruitment chains and participant as the second level cluster to account for within-participant correlations. Odds ratios were calculated. White ethno-racial identity was used as the initial reference group, as most participants identified as white and given the social privilege ascribed to this group. We completed additional analyses using Asian identity (the second most common identity before RDS adjustment) as the reference group. Variables included for consideration in the multivariable model were selected based on *p*-value < 0.2 in the univariable model and/or previous research. The final models were selected using a backward stepwise selection technique based on two criteria (Akaike Information Criterion (AIC) and Type III *p*-values), whereby the least significant (i.e., highest Type III *p*-value) variable was dropped until the final models reached the optimal (minimum) AIC [37]. All analyses were performed using SAS® Version 9.4 (SAS Corporation Cary, North Carolina, United States). All statistical tests were two-sided and considered significant at *p* < 0.05.

## Results

A total of 774 individuals were enrolled between February 2012 and February 2015 including 134 (17.3%) recruited as initial seeds. All 774 contributed at least one observation to the study and 583 (75.3%) completed at least one follow-up visit by February 2017. The median follow-up time was 3.4 years (Q1-Q3: 2.5—3.5), and the median number of follow-up visits was 6 (Q1-Q3: 4–7). Of the 774 participants with at least one study visit, the median number of follow-up visits did not differ across ethno-racial categories groups, (*p* = 0.241). After RDS adjustment, 34.4% of the sample were aged < 30 years, 79.9% identified as gay, and 20.1% identified as bisexual or other. The majority of respondents (67.4%) reported some post-secondary education, while 72.9% reported an annual income less than $30,000. Over one in four participants (26.6%) reported not being born in Canada. Almost a quarter (21.4%) self-reported as living with HIV, and 70.2% reported being “out” about their sexual orientation. In terms of ethno-racial identity groups, 68.6% identified as white, 9.2% as Asian, 9.8% as Indigenous (Indigenous), 7.3% as Latino, and 5.1% as other racial or ethnic identities. At enrollment, 27.7% (95% CI: 22.5–32.7%) had moderate/severe HADS anxiety scores and 5.9% (95% CI: 3.2–9.4%) had moderate/severe depression scores.

RDS-adjusted descriptive statistics by ethno-racial identity group are shown in Table 1. We found statistically significant differences (*p* < 0.001) for at least one group for all sociodemographic factors and substance use measures. However, we did not find differences between groups for measures of loneliness (*p* = 0.994), overall self-esteem (*p* = 0.376), or collective self-esteem (*p* = 0.541). We found differences in the distribution of normal, borderline, and moderate/severe depression scores at enrollment across ethno-racial groups (*p* = 0.003), but the differences in anxiety scores did not reach statistical significance (*p* = 0.067).

**Table 1:** RDS adjusted descriptive statistics of gbMSM living in Metro Vancouver, Canada at enrollment (*n* = 774)

	**White** (*n* = 585; RDS% = 68.6%)			**Asian** (*n* = 74; RDS % = 9.2%)			**Indigenous** (*n* = 50; RDS % = 9.8%)			**Latino** (*n* = 35; RDS % = 7.3%)			**Other** (*n* = 30; RDS % = 5.1%)			
	**n**	**RDS %**	**RDS 95% CI**	**n**	**RDS %**	**RDS 95% CI**	**n**	**RDS %**	**RDS 95% CI**	**n**	**RDS %**	**RDS 95% CI**	**n**	**RDS %**	**RDS 95% CI**	***P*-Value**
Demographics																
**Income Group**																
Less than $30,000	356	67.4	62.2, 72.6	39	61.5	48.1, 74.9	46	95.0	89.0, 100.0	27	86.9	74.5, 99.2	17	55.9	29.2, 82.6	< 0.001
$30,000—$59,999	161	22.6	18.0, 27.1	21	21.4	10.9, 31.8	4	5.0	0.0, 11.0	4	6.4	0.0, 14.8	10	35.3	9.6, 61.0	
$60,000 and over	68	10.0	6.8, 13.2	14	17.1	7.6, 26.7	0	0.0	0.0, 0.0	4	6.7	0.0, 15.8	3	8.8	0.0, 21.2	
**Education completed**																
High school or less	137	29.7	24.0, 35.4	3	7.4	0.0, 16.1	26	54.4	33.6, 75.1	7	18.0	0.2, 35.8	6	26.3	0.6, 52.1	< 0.001
Greater than high school	448	70.3	64.6, 76.0	71	92.6	83.9, 100.0	24	45.6	24.9, 66.4	28	82.0	64.2, 99.8	24	73.7	47.9, 99.4	
**Born in Canada**																
No	82	14.3	10.2, 18.4	52	76.7	65.9, 87.5	0	0.0	0.0, 100	31	95.4	90.1, 100.0	12	51.9	24.9, 78.8	< 0.001
Yes	503	85.7	81.6, 89.8	22	23.3	12.5, 34.1	50	100.0	0.0, 100	4	4.6	0.0, 9.9	18	48.1	21.2, 75.1	
**Being Out**																
Partially/no	37	8.2	4.8, 11.6	24	39.0	23.8, 54.2	5	5.3	0.0, 10.9	5	20.7	1.6, 39.8	1	6.4	0.0, 19.3	< 0.001
Yes	479	77.2	72.1, 82.2	47	55.0	39.9, 70.1	37	70.1	50.9, 89.4	26	64.6	41.7, 87.4	22	57.7	29.5, 85.9	
Not gay-identified	69	14.6	10.4, 18.8	3	6.0	0.0, 13.8	8	24.5	5.6, 43.5	4	14.7	0.0, 31.9	7	35.9	7.3, 64.5	
Substance Use, P6M																
**P6M Crystal Methamphetamine**																
No	469	81.8	77.2, 86.4	70	95.6	90.2, 100.0	27	58.8	38.9, 78.8	30	86.9	70.1, 100.0	24	90.5	79.8, 100.0	< 0.001
Yes	116	18.2	13.6, 22.8	4	4.4	0.0, 9.8	23	41.2	21.2, 61.1	5	13.1	0.0, 29.9	6	9.5	0.0, 20.2	
**P6M Crack**																
No	536	89.6	85.3, 93.9	71	95.5	89.9, 100.0	34	58.7	37.6, 79.8	33	91.8	75.9, 100.0	26	80.0	55.7, 100.0	< 0.001
Yes	49	10.4	6.1, 14.7	3	4.5	0.0, 10.1	16	41.3	20.2, 62.4	2	8.2	0.0, 24.1	4	20.0	0.0, 44.3	
**AUDIT Zone**																
Low Risk	351	63.5	58.1, 69.0	55	82.7	73.6, 91.7	17	28.7	9.3, 48.0	21	63.6	40.9, 86.3	17	67.1	41.5, 92.8	< 0.001
Medium Risk	162	25.8	20.9, 30.7	14	11.8	4.4, 19.3	9	20.7	4.2, 37.2	10	24.4	5.0, 43.8	9	26.4	1.4, 51.3	
Harmful	37	5.1	3.1, 7.0	2	2.8	0.0, 6.7	13	22.3	6.0, 38.7	3	3.8	0.0, 9.9	1	0.9	0.0, 2.7	
Possible Dependence	33	5.6	2.9, 8.3	3	2.7	0.0, 6.3	9	28.3	7.4, 49.2	1	8.2	0.0, 24.1	2	5.6	0.0, 15.0	
Mental Health																
**HADS-Anxiety Scores**																
Normal (0–7)	268	42.1	36.4, 47.7	45	57.1	43.2, 71.1	24	53.6	32.4, 74.7	19	49.1	25.2, 72.9	17	44.4	17.5, 71.3	0.067
Mild (8–10)	156	27.7	22.4, 33.1	17	27.9	14.6, 41.3	11	20.4	4.2, 36.5	9	33.8	9.9, 57.8	7	31.5	5.1, 57.8	
Moderate/severe (> 10)	157	30.2	24.7, 35.7	10	14.9	5.2, 24.6	13	26.1	6.9, 45.3	7	17.1	0.0, 34.8	5	24.1	0.0, 49.3	
**HADS Depression Scores**																
Normal (0–7)	486	82.5	77.9, 87.1	66	91.7	83.9, 99.6	40	85.5	70.4, 100.0	31	86.1	68.8, 100.0	26	72.3	42.4, 100.0	0.003
Mild (8–10)	59	12.1	7.9, 16.3	5	7.5	0.0, 15.2	4	2.6	0.0, 6.1	4	13.9	0.0, 31.2	2	14.8	0.0, 39.1	
Moderate/severe (> 10)	36	5.4	3.1, 7.6	1	0.8	0.0, 2.4	4	11.9	0.0, 26.8	0	0.0	0.0, 0.0	1	12.9	0.0, 37.3	
	**MD**	**RDS MD**	**RDS Q1, Q3**	**MD**	**RDS MD**	**RDS Q1, Q3**	**MD**	**RDS MD**	**RDS Q1, Q3**	**MD**	**RDS MD**	**RDS Q1, Q3**	**MD**	**RDS MD**	**RDS Q1, Q3**	
**Age**	34	34	25, 49	30	29	23, 38	37	37	30, 44	31	30	24, 38	33	36	29	0.003
**Self Esteem Total Score**	7	7	4, 10	7	7	5, 9	7	7	4, 10	5	7	3, 7	6	7	4, 9	0.376
**Collective Self Esteem Score**	8	7	6, 9	8	8	6, 9	8	8	7, 9	7	7	6, 8	8	7	6, 10	0.541
**Loneliness Score**	2	3	1, 5	2	3	1, 5	2	3	1, 4	2	3	1, 4	2	4	1, 5	0.994

*RDS* Respondent Driven Sampling, *95% CI* 95% confidence interval, *P6M* Past 6 months, *AUDIT* Alcohol Use Disorders Identification Test, *HAD* Hospital Anxiety & Depression Scale, *MD* Median, *Q1,Q3* First quartile, third quartile values

In the univariable analysis of factors associated with moderate/severe HADS anxiety scores, participants who identified as Asian (OR = 0.31; 95% CI: 0.13–0.77) and Latino (OR = 0.23; 95% CI: 0.05–0.97) had decreased odds of moderate/severe anxiety scores compared with white participants (Table 2). This association remained in the multivariable model for Asian participants (aOR = 0.39; 95% CI: 0.18–0.86) but was not retained for Latino participants (aOR = 0.39; 95% CI: 0.09–1.73). We found no other significant differences between ethno-racial groups when changing the reference group to from white to Asian (data not shown). Other factors associated with increased odds of moderate/severe anxiety score in the multivariable model included AUDIT Score (aOR = 1.06; 95% CI: 1.02–1.09), total self-esteem score (aOR = 1.43; 95% CI: 1.33–1.53), and loneliness score (aOR = 1.67; 95% CI: 1.50–1.86). Increasing age was associated with a lower odd of moderate/severe scores (aOR = 0.98 per year; 95% CI: 0.96–1.00).

**Table 2:** Univariable and multivariable generalized linear mixed models of factors associated with HADS **anxiety** score > 10 among gbMSM living in Metro Vancouver, Canada

	**Univariable**		**Multivariable**	
	**OR**	**95% CI**	**aOR**	**95% CI**
**Age**	**0.98**	**0.96, 1.00**	**0.98**	**0.96, 1.00**
**AUDIT Score**	**1.10**	**1.05, 1.14**	**1.06**	**1.02, 1.09**
**Self Esteem scale total score**	**1.64**	**1.53, 1.75**	**1.43**	**1.33, 1.53**
**Collective Self Esteem Score**	**0.92**	**0.85, 1.00**	Not Selected	
**Loneliness score**	**2.23**	**2.00, 2.49**	**1.67**	**1.50, 1.86**
**Ethno-racial identity**				
White	Ref			
Asian	**0.31**	**0.13, 0.77**	**0.39**	**0.18, 0.86**
Indigenous	1.13	0.32, 3.97	0.79	0.29, 2.10
Latino	**0.23**	**0.05, 0.97**	0.39	0.09, 1.73
Other	0.38	0.10, 1.53	0.59	0.15, 2.36
**Income group**				
Less than $30,000	Ref			
$30,000—$59,999	0.76	0.50, 1.16	Not Selected	
$60,000 and over	**0.52**	**0.30, 0.92**		
**Education**				
High school or less	Ref			
Greater than high school	**0.52**	**0.29, 0.94**	Not Selected	
**Born in Canada**				
No	Ref			
Yes	**2.51**	**1.28, 4.92**	Not Selected	
**Immigration status**				
Born in Canada	Ref			
Canadian Citizen/Permanent Resident	**0.46**	**0.23.0.94**	Not Selected	
Temporary Status/Refugee/Other	**0.23**	**0.08,0.72**		
**Current Housing**				
Stable	Ref			
Unstable	1.41	0.86, 2.31	Not Selected	
**Being Out**				
Partially/no	Ref			
Yes	0.96	0.46, 2.02	Not Selected	
Not gay-identified	1.29	0.52, 3.23		
**Sexual Identity**				
Gay	Ref			
Bisexual/Other	1.48	0.85, 2.58	Not Selected	
**Self-Reported HIV Status**				
HIV Negative/Unknown	Ref			
HIV Positive	1.08	0.58, 2.01	Not Selected	
**P6M Crystal**				
No	Ref			
Yes	**2.28**	**1.43, 3.65**	Not Selected	
**P6M Crack**				
No	Ref			
Yes	**3.87**	**1.74, 8.61**	1.71	0.77, 3.77

Data with bold emphasis indicates statistical significance at *p* < .05

*HAD* Hospital Anxiety & Depression Scale, *OR* Odds ratio, *95% CI* 95% confidence interval, *AUDIT* Alcohol Use Disorders Identification Test, *P6M* Past 6 months

In univariable analyses of factors associated with moderate/severe HADS depression scores, Latino identity was significantly associated with decreased odds of having moderate/severe HADS depression scores compared to both white (OR = 0.01; 95% CI: 0.01–0.03) (Table 3) and Asian (OR = 0.03; 95% CI: 0.01–0.06) identity (data not shown). This association was retained in the multivariable model for Latino individuals in comparison with both white (aOR = 0.17; 95% CI: 0.08–0.36) and Asian (aOR = 0.07; 95% CI: 0.02–0.20) individuals. No other significant differences were found between ethno-racial groups when we changed the reference category as above (data not shown). Other factors associated with increased odds of moderate/severe depression scores in our multivariable analysis included age (aOR = 1.06; 95% CI: 1.03–1.08), self-esteem score (aOR = 1.55; 95% CI: 1.44–1.67), loneliness score (aOR = 1.78; 95% CI: 1.51–2.11) and unstable housing (aOR = 2.16; 95% CI: 1.19–3.95).

**Table 3:** Univariable and multivariable generalized linear mixed models of factors associated with HADS depression score > 10 among gbMSM living in Metro Vancouver, Canada

	**Univariable**		**Multivariable**	
	**OR**	**95% CI**	**aOR**	**95% CI**
**Age**	**1.04**	**1.02, 1.06**	**1.06**	**1.03, 1.08**
**AUDIT Score**	1.05	0.99, 1.10	Not selected	
**Self Esteem scale total score**	**1.74**	**1.58, 1.91**	**1.55**	**1.44, 1.67**
**Collective Self Esteem Score**	0.89	0.79, 1.02	Not selected	
**Loneliness score**	**2.62**	**2.21, 3.11**	**1.78**	**1.51, 2.11**
**Ethno-racial identity**				
White	Ref			
Asian	0.64	0.29, 1.41	2.23	0.76, 6.54
Indigenous	1.86	0.72, 4.84	1.18	0.30, 4.66
Latino	**0.01**	**0.01, 0.03**	**0.17**	**0.08, 0.36**
Other	0.74	0.18, 3.00	2.19	0.27, 17.65
**Income group**				
Less than $30,000	Ref			
$30,000—$59,999	0.84	0.43, 1.61	Not Selected	
$60,000 and over	0.40	0.12, 1.34	Not Selected	
**Education**				
High school or less	Ref			
Greater than high school	**0.26**	**0.16, 0.42**	0.54	0.27, 1.08
**Born in Canada**				
No	Ref			
Yes	1.45	0.79, 2.67	Not Selected	
**Immigration status**				
Born in Canada	Ref			
Canadian Citizen/ Permanent Resident	**0.93**	**0.48, 1.78**	Not Selected	
Temporary Status/Refugee/Other	0.17	0.03,1.15		
**Current Housing**				
Stable	Ref			
Unstable	**3.17**	**1.71, 5.87**	**2.16**	**1.19, 3.95**
**Being Out**				
Partially/no	Ref			
Yes	1.11	0.30, 4.12	Not Selected	
Not gay-identified	2.42	0.61, 9.56	Not Selected	
**Sexual Identity**				
Gay	Ref			
Bisexual/Other	1.45	0.65, 3.26	Not Selected	
**Self-Reported HIV Status**				
HIV Negative / Unknown	Ref			
HIV Positive	**2.50**	**1.40, 4.45**	Not Selected	
**P6M Crystal**				
No	Ref			
Yes	**2.25**	**1.01, 4.99**	Not Selected	
**P6M Heroin**				
No	Ref			
Yes	**6.16**	**1.30, 29.28**	Not Selected	

Data with bold emphasis indicates statistical significance at *p* < .05

*HAD* Hospital Anxiety & Depression Scale, *OR* Odds ratio, *95% CI* 95% confidence interval, *AUDIT* Alcohol Use Disorders Identification Test, *P6M* Past 6 months

## Discussion

In a longitudinal analysis of 774 sexually-active gbMSM in Vancouver, Canada, we did not find evidence of increased odds of moderate/severe anxiety or depression scores amongst ethno-racial minority men compared to white men. In fact, we found reduced odds of anxiety symptoms for Asian men and depressive symptoms for Latino men when compared to their white counterparts.

Our results are consistent with research in general populations in the United States and United Kingdom, which found similar or lower levels of mental health symptoms, suicidal ideation, or doctor-diagnosed mental health conditions among Black, Hispanic, and Asian individuals compared with white individuals [13, 14, 23]. This has similarly been demonstrated in some large-scale national studies in sexual minority populations in the U.S. and U.K. [3, 15, 17, 18]. Notably, in the U.S. National Latino and Asian American Survey conducted in 2002–2003, Latino and Asian sexual minority individuals had lower levels of depressive, anxiety, substance use, and eating disorders, identified through diagnostic interview, than comparable studies of sexual minorities in general [15]. However, some findings in the extant literature varied with differing mental health indicators (i.e., suicidality) or when evaluating non-white identities individually rather than a composite group. For example, although it was found that non-white lesbian and gay adolescents overall had similar or decreased levels of depressive symptoms, this differed between Black and Asian as compared to Latino, multiracial, and American Native/Pacific Islander youth, the latter ethno-racial groups experiencing increased depressive symptoms [17, 20]. Additionally, some U.S. research has found increased levels of suicidality or suicide attempts among sexual minority people of colour [16, 19, 20]. Indeed, research in the U.S. and Canada has shown that members of ethnic minorities with mental health symptoms or disorders are more likely to have persistent disorders [13], more likely to have unmet mental health care needs [23, 38], and less likely to visit mental health resources [23].

Several hypotheses exist to explain the similar or reduced rates of mental health symptoms among gbMSM of colour, including immigration status, family cohesion, and resilience or stress-related growth. In our study, Latino and Asian men were significantly less likely to be born in Canada. In North America, and specifically in Canada, some research has shown evidence of a “healthy immigrant” effect, at least initially, resulting in better mental health outcomes for immigrants [12]. Family cohesion and support, which may be higher in Latino and Asian communities [39], may also serve as a protective factor against poor mental health outcomes [40]. Finally, the previous experiences of gbMSM of colour may contribute to their resilience against sexual minority-related stigma and discrimination. For example, members of racial and ethnic minority groups can learn mechanisms for coping with minority stress in childhood through family members and close adult figures [31]. GbMSM of color may also acquire coping skills earlier in life through minority stress-related experiences associated with an ethno-racial minority identity [9, 41]. Taken together, these factors may explain the reduced levels of anxiety or depression symptoms among Asian and Latino men in our study.

Some differences in mental health symptoms may also be explained through other factors in the multivariable analysis. We selected variables in our multivariable analysis based on factors found in the literature that may confound mental health outcomes. For example, differences were found in alcohol use (AUDIT score) between ethno-racial groups, with a significant association between AUDIT score and anxiety symptoms. Alcohol use is often comorbid with anxiety [42] or depression [43] symptoms, with a potential causal relationship between alcohol use disorder and major depression [43]. Additionally, significant associations were found between loneliness and self-esteem scores and anxiety and depression symptoms, although differences were not found between groups. Finally, significant differences between ethno-racial groups were found in income and education level, which are important social determinants of mental health that have been found to be associated with poor mental health outcomes or mental health disorders [44]. These variables were largely associated with symptom scores in the univariable analyses but were not retained in our multivariable model. Thus, given that the decreased odds of moderate/severe anxiety scores for Latino men was not retained in the multivariable model, other variables may be involved that may serve as the topic of further research.

A key strength of our study was the use of respondent-driven sampling (RDS), with the inclusion of ethno-racial minority seeds, as our sampling and analysis methodology. RDS has the potential to overcome previous sampling shortfalls and more accurately represent population parameters for the Metro Vancouver gbMSM population. Additionally, it allows for the inclusion of subgroups and communities that may be missed through other recruiting methods and enables the study team to adjust for some known biases that arise through the recruitment process. Our longitudinal analysis also increases our statistical power to examine associations between smaller ethno-racial groups while accounting for non-independence of repeated measures and recruitment chains. This allowed our study to address the limitation of relatively small numbers of participants who identified as racial or ethnic minorities. Additionally, our sample was drawn from gbMSM residing in Metro Vancouver, Canada, and thus may not reflect either rural or other urban contexts elsewhere. Greater Vancouver contains a greater proportion of visible minority individuals than Canada as a whole [45], and research has shown a protective effect of higher immigrant concentration neighborhoods on mental health disorder prevalence for immigrants in Canada [12]. Finally, we used the HADS, which utilizes previously established and validated cut-offs, to measure depression and anxiety symptoms [46]. However, given the limited sample of individuals with moderate/severe depression, scores, this may potentially reduce our power to detect significant effects. Additionally, more research is needed to determine the validity of the HADS scale within ethno-racial minority populations in Canada and the United States.

Further research is needed to understand the distinct social, cultural, and structural mechanisms that modulate mental health for gbMSM of various ethno-racial identities. Moving forward, an important research objective remains in understanding survival and resilience narratives that promote mental health among gbMSM of colour. Healthcare providers should nevertheless be aware of the unique mechanisms of racism and heterosexism interact when working with gbMSM.

## Conclusion

Among a sample of urban-residing gbMSM in Vancouver, Canada, ethno-racial minority status was not associated with increased odds of moderate/severe depression or anxiety symptoms. Asian and Latino gbMSM in Metro Vancouver manage multiple minority stressors without higher self-reported burden of mental health symptoms. Of note, gbMSM still experience a higher burden of mental health disorders than the general population in Canada. As such, more research is needed with ethno-racialized minority gbMSM to understand mental health risks and vulnerabilities and to qualify survival and resilience narratives surrounding mental health. Healthcare providers should be aware of the complexity in how racism and heterosexism interact when working with gbMSM.

## Data Availability

The datasets generated and/or analysed during the current study are not publicly available due to our ethics approvals and privacy policies. However, the British Columbia Centre for Excellence in HIV/AIDS (BC-CfE) may, upon meeting all legislative and policy obligations, provide de-identified data used in the manuscript for external research use. Requests for access to the research dataset must be directed to the Privacy Officer at privacy@bccfe.ca. If approved, the de-identified research dataset will be provided via Secure File Transfer Protocol to the requesting researcher.

## References

[1] Brennan DJ, Ross LE, Dobinson C, Veldhuizen S, Steele LS (2010). Men’s sexual orientation and health in Canada. Can J Public Heal.

[2] Chakraborty A, McManus S, Brugha TS, Bebbington P, King M (2011). Mental health of the non-heterosexual population of England. Br J Psychiatry.

[3] Rodriguez-Seijas C, Eaton NR, Pachankis JE (2019). Prevalence of psychiatric disorders at the intersection of race and sexual orientation: Results from the National Epidemiologic Survey of Alcohol and Related Conditions-III. J Consult Clin Psychol.

[4] Meyer IH (2003). Prejudice, social stress, and mental health in lesbian, gay, and bisexual populations: conceptual issues and research evidence. Psychol Bull.

[5] Meyer IH (1995). Minority stress and mental health in gay men. J Health Soc Behav.

[6] Herek GM, Gillis JR, Cogan JC (1999). Psychological sequelae of hate-crime victimization among lesbian, gay, and bisexual adults. J Consult Clin Psychol.

[7] Paradies Y, Ben J, Denson N, Elias A, Priest N, Pieterse A (2015). Racism as a determinant of health: A systematic review and meta-analysis. PLoS ONE.

[8] Crenshaw K (1991). Mapping the margins: intersectionality, identity politics, and violence against women of color. Stanford Law Rev.

[9] Meyer IH (2010). Identity, stress, and resilience in lesbians, gay men, and bisexuals of color. Couns Psychol.

[10] Cyrus K (2017). Multiple minorities as multiply marginalized: applying the minority stress theory to LGBTQ people of color. J Gay Lesbian Ment Heal.

[11] Center for Behavioral Health Statistics and Quality. Racial/Ethnic Differences in Substance Use, Substance Use Disorders, and Substance Use Treatment Utilization among People Aged 12 or Older (2015-2019). Substance Abuse and Mental Health Services Administration; 2021. https://www.samhsa.gov/data/sites/default/files/reports/rpt35326/2021NSDUHSUChartbook.pdf.

[12] Menezes NM, Georgiades K, Boyle MH (2011). The influence of immigrant status and concentration on psychiatric disorder in Canada: A multi-level analysis. Psychol Med.

[13] Breslau J, Kendler KS, Su M, Gaxiola-Aguilar S, Kessler RC (2005). Lifetime risk and persistence of psychiatric disorders across ethnic groups in the United States. Psychol Med.

[14] Ortega AN, Rosenheck R, Alegría M, Desai RA (2000). Acculturation and the lifetime risk of psychiatric and substance use disorders among Hispanics. J Nerv Ment Dis.

[15] Cochran SD, Mays VM, Alegria M, Ortega AN, Takeuchi D (2007). Mental health and substance use disorders among Latino and Asian American lesbian, gay, and bisexual adults. J Consult Clin Psychol.

[16] Meyer IH, Dietrich J, Schwartz S (2008). Lifetime prevalence of mental disorders and suicide attempts in diverse lesbian, gay, and bisexual populations. Am J Public Health.

[17] Kiekens WJ, La Roi C, Dijkstra JK (2020). Sexual identity disparities in mental health among U.K. adults, U.S. adults, and U.S. adolescents: examining heterogeneity by race/ethnicity. Psychol Sex Orientat Gend Divers.

[18] Almazan EP (2019). Are Black sexual minority adults more likely to report higher levels of psychological distress than white sexual minority adults? Findings from the 2013–2017 National Health Interview Survey. Soc Sci.

[19] Remafedi G (2002). Suicidality in a venue-based sample of young men who have sex with men. J Adolesc Heal.

[20] Bostwick WB, Meyer I, Aranda F, Russell S, Hughes T, Birkett M (2014). Mental health and suicidality among racially/ethnically diverse sexual minority youths. Am J Public Health.

[21] Prestage G, Hammoud M, Jin F, Degenhardt L, Bourne A, Maher L (2018). Mental health, drug use and sexual risk behavior among gay and bisexual men. Int J Drug Policy.

[22] Pakula B, Shoveller J, Ratner PA, Carpiano R (2016). Prevalence and co-occurrence of heavy drinking and anxiety and mood disorders among gay, Lesbian, bisexual, and heterosexual Canadians. Am J Public Health.

[23] Chiu M, Amartey A, Wang X, Kurdyak P (2018). Ethnic differences in mental health status and service utilization: a population-based study in Ontario. Canada Can J Psychiatry.

[24] Gilmour H (2014). Positive mental health and mental illness. Heal Rep.

[25] Heckathorn DD (1997). Respondent-driven sampling: A new approach to the study of hidden populations. Soc Probl.

[26] Heckathorn DD (2002). Respondent-driven sampling II: Deriving valid population estimates from chain-referral samples of hidden populations. Soc Probl.

[27] Magnani R, Sabin K, Saidel T, Heckathorn D (2005). Review of sampling hard-to-reach and hidden populations for HIV surveillance. AIDS, Suppl.

[28] Moore DM, Cui Z, Lachowsky N, Raymond HF, Roth E, Rich A (2016). HIV community viral load and factors associated with elevated viremia among a community-based sample of men who have sex with men in Vancouver. Canada J Acquir Immune Defic Syndr.

[29] Stern AF (2014). The hospital anxiety and depression scale. Occup Med (Lond).

[30] Brennan C, Worrall-Davies A, McMillan D, Gilbody S, House A (2010). The hospital anxiety and depression scale: a diagnostic meta-analysis of case-finding ability. J Psychosom Res.

[31] Herek GM, Glunt EK. Identity and community among gay and bisexual men in the aids era: preliminary findings from the Sacramento men's health study. In: AIDS, Identity, and Community: The HIV Epidemic and Lesbians and Gay Men. Vol. 2. Thousand Oaks: SAGE Publications, Inc.; 1995. p. 55–84.

[32] Lubben J, Blozik E, Gillmann G, Iliffe S, Von Kruse WR, Beck JC (2006). Performance of an abbreviated version of the lubben social network scale among three European community-dwelling older adult populations. Gerontologist.

[33] de Jong Gierveld J, van Tilburg T (2006). A 6-item scale for overall, emotional and social loneliness: confirmatory tests on survey data. Res Aging.

[34] Lai HMX, Cleary M, Sitharthan T, Hunt GE (2015). Prevalence of comorbid substance use, anxiety and mood disorders in epidemiological surveys, 1990–2014: a systematic review and meta-analysis. Drug Alcohol Depend.

[35] Quello SB, Brady KT, Sonne SC (2005). Mood disorders and substance use disorder: a complex comorbidity. Sci Pract Perspect.

[36] Saunders JB, Aasland OG, Babor TF, De La Fuente JR, Grant M (1993). Development of the Alcohol Use Disorders Identification Test (AUDIT): WHO Collaborative Project on Early Detection of Persons with Harmful Alcohol Consumption-II. Addiction.

[37] Lima VD, Geller J, Bangsberg DR, Patterson TL, Daniel M, Kerr T (2007). The effect of adherence on the association between depressive symptoms and mortality among HIV-infected individuals first initiating HAART. AIDS.

[38] Wells K, Klap R, Koike A, Sherbourne C (2001). Ethnic disparities in unmet need for alcoholism, drug abuse, and mental health care. Am J Psychiatry.

[39] Ruiz ME (2007). Familismo and filial piety among Latino and Asian elders: Reevaluating family and social support. Hisp Heal Care Int.

[40] Leong F, Park YS, Kalibatseva Z (2013). Disentangling immigrant status in mental health: Psychological protective and risk factors among latino and asian american immigrants. Am J Orthopsychiatry.

[41] Vaughn AA, Roesch SC, Aldridge AA (2009). Stress-related growth in racial/ethnic minority adolescents: Measurement structure and validity. Educ Psychol Meas.

[42] Schuckit MA, Hesselbrock V (1994). Alcohol dependence and anxiety disorders: what is the relationship?. Am J Psychiatry.

[43] Boden JM, Fergusson DM (2011). Alcohol and depression. Addiction.

[44] Silva M, Loureiro A, Cardoso G (2016). Social determinants of mental health: A review of the evidence. Eur J Psychiatry.

[45] Government of Canada, Statistics Canada. The Daily — Immigration and ethnocultural diversity: key results from the 2016 census. Statistics Canada; 2016. https://www150.statcan.gc.ca/n1/daily-quotidien/171025/dq171025b-eng.htm.

[46] Bjelland I, Dahl AA, Haug TT, Neckelmann D (2002). The validity of the hospital anxiety and depression scale. J Psychosom Res.

